# High-Endurance, Low-Loss Sb_2_Se_3_ Optical Switches on Silicon Nitride Using Transparent Conductive Heaters

**DOI:** 10.1021/acsphotonics.6c01756

**Published:** 2026-09-25

**Authors:** Xingshi Yu, Ipsita Chakraborty, Isaac Johnson, Savvas I. Raptis, Qianbin Luo, Hao Liu, Thalia D. Bucio, Elliot Sandell, Christos Vagionas, Amalia Miliou, Periklis Petropoulos, Nikos Pleros, Ioannis Zeimpekis, Frederic Gardes

**Affiliations:** † Optoelectronics Research Centre (ORC), 7423 University of Southampton, University Road, Southampton, SO17 1BJ Hampshire, U.K.; ‡ Wireless and Photonic Systems and Networks (WinPhoS) research group, 37782 The Aristotle University of Thessaloniki, 3is Septemvriou Street, Agios Dimitrios, Thessaloniki 54124, Greece; § Electronics and Computer Science (ECS), University of Southampton, University Road, Southampton, SO17 1BJ Hampshire, U.K.

**Keywords:** phase-change materials, transparent ITO heater, silicon nitride photonics, nonvolatile optical switching, high endurance, multilevel photonic memory

## Abstract

We report an electrically actuated, low-loss nonvolatile optical switch based on the phase-change material (PCM) Sb_2_Se_3_ integrated on a silicon nitride (Si_3_N_4_) platform. The device is fabricated using an 8-in. Wafer-scale process flow, demonstrating the feasibility of scalable manufacturing for photonic integrated circuits (PICs). By employing adaptive voltage and transparent indium tin oxide (ITO) microheaters, reversible switching between the amorphous and crystalline states is achieved with an endurance exceeding 140 million switching cycles, establishing a new benchmark for nonvolatile integrated photonic memory and reconfigurable architectures. Furthermore, multilevel operation beyond 6 bits can be repeatably demonstrated by pulse-count-dependent gradual crystallization, enabling precise control of the optical phase. These results highlight a scalable and energy-efficient platform for high-density programmable and nonvolatile photonic integrated systems.

## Graphical abstract

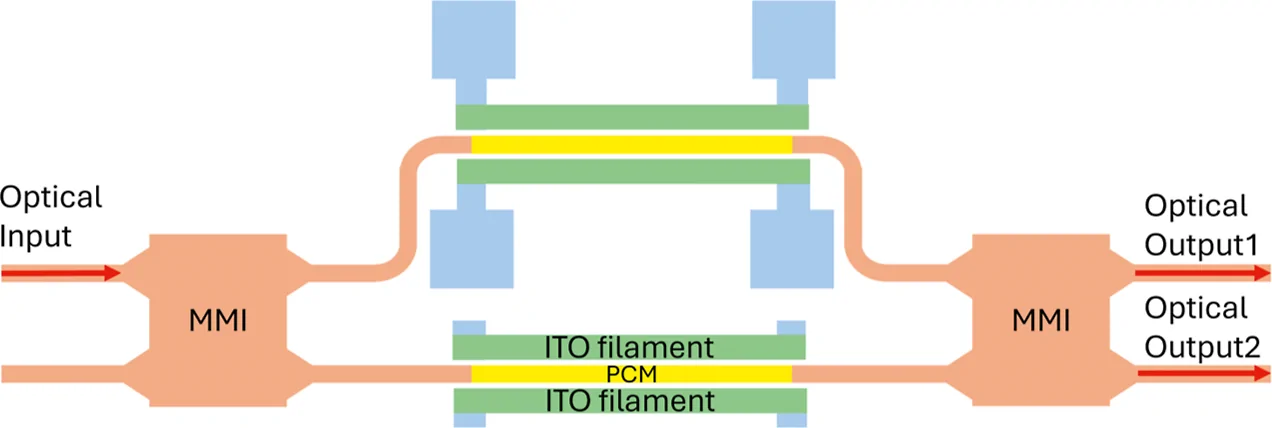

## Introduction

Silicon nitride (Si_3_N_4_) provides a low-loss platform for integrating photonic components. Its low thermo-optic coefficient enhances thermal stability, while its moderate refractive index contrast improves fabrication tolerance.^1,2^ These features support scalable, high-yield photonic integrated circuits (PICs) for next-generation data centers and high-performance computing.^3–5^ Central to these systems of applications are phase shifters, enabling dynamic signal routing, reconfigurable multiplexing, and programmable photonic functionalities.^6–8^ Conventional implementations based on thermo-optic or carrier-induced plasma dispersion effects are inherently volatile, requiring continuous electrical bias to maintain a programmed state.^9–12^

The integration of nonvolatile phase-change materials (PCMs) provides a promising route to overcome this limitation.^13^ Sb_2_Se_3_ exhibits low absorption while providing reversible refractive index changes between amorphous and crystalline phases, enabling zero-static-power optical switching.^14–17^ These transitions can be triggered optically or electrically, supporting applications in photonic memory, neuromorphic computing, and optical field-programmable gate arrays (FPGA).^18–21^ On-chip resistive heaters allow compact integration and compatibility with established electronic packaging processes. Compared with conventional metallic heaters, transparent conductive materials with lower refractive index, such as indium tin oxide (ITO), are widely adopted to further minimize optical loss arising from substantial parasitic metallic absorption.^22^

We present a high-endurance nonvolatile optical switch based on the heterogeneous integration of Sb_2_Se_3_ on a Si_3_N_4_ platform. We utilize ITO as a transparent resistive microheater to trigger the phase transition. This configuration enables low-loss, efficient, localized Joule heating, facilitating reversible switching between the amorphous and crystalline states of the Sb_2_Se_3_ film.^15,22^ The nonvolatile photonic switch is designed using a Mach–Zehnder interferometer (MZI) configuration. In Figure 1, the photonic circuitry utilizes Si_3_N_4_ waveguides with width and thickness of 1 μm × 400 nm respectively, optimized for single-mode operation of the fundamental transverse electric (TE) mode at telecommunication wavelengths.^23^ The active switching mechanism is based on the phase transition of a 20 nm thick Sb_2_Se_3_ film.^24,25^ This PCM is deposited on a 20 nm thick SiO_2_ optical spacer, which serves as a critical buffer layer to minimize surface scattering and protect the waveguide core from interfacial diffusion during thermal processes.^26,27^ To suppress oxidation of PCM and improve thermal stability during cycling, a 40 nm ZnS/SiO_2_ (20%:80%) cladding layer is used.^28^ The relatively low thermal conductivity helps balance the thermal conditions required for amorphization and crystallization.^29^ A key design feature is the 300 nm lateral gap between the Si_3_N_4_ waveguide core and the integrated ITO microheater filaments. Electro-thermal and optical cosimulations indicate that, at this separation, the evanescent field of the fundamental TE mode decays sufficiently, limiting optical absorption from the ITO elements to below 0.005 *d*B/μm, while maintaining efficient heat transfer. Based on four-probe measurements, the sheet resistance (*R*_sh_) of the in-house deposited ITO layer was ascertained to be 17 Ω/sq. To ensure compatibility with standard copackaging optics (CPO) drivers, the operating voltage was maintained below ∼10 V by fixing the filament width at 5 μm,^30^ while scaling its length with the PCM region. The detailed fabrication processes are shown in Support Information Section S1.

**Figure 1:**
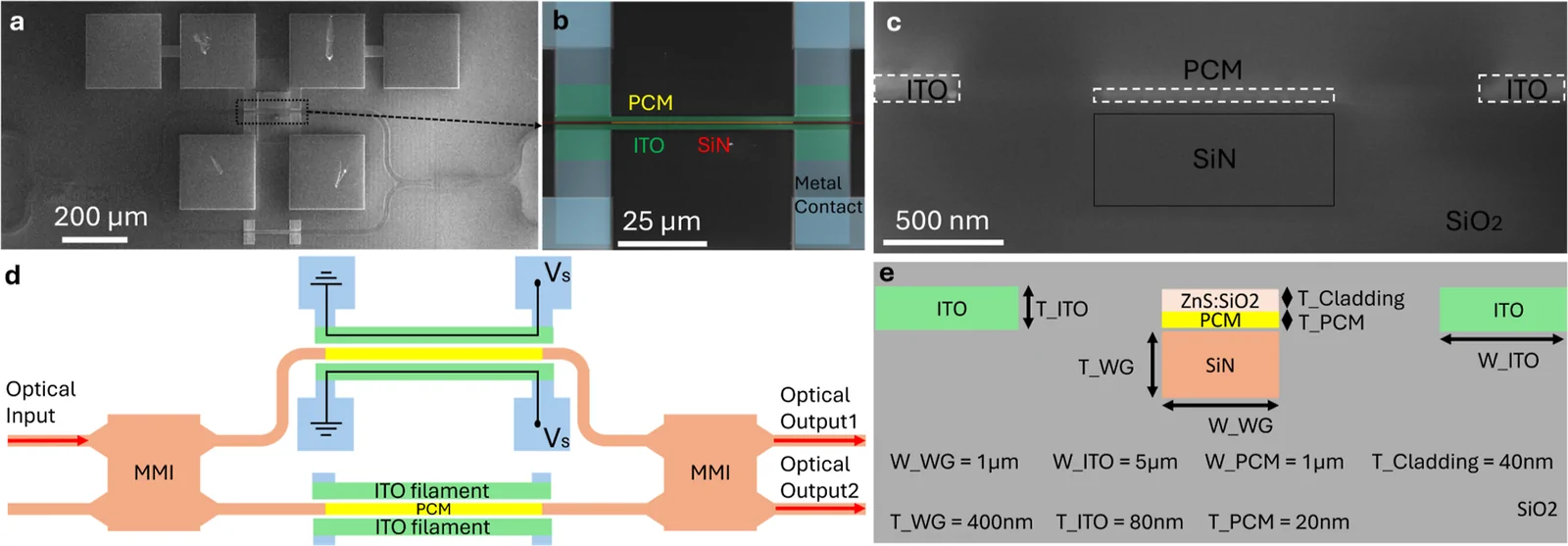
Device design and structural characterization of the MZI with an integrated PCM phase shifter. (a) Top-view SEM image of the fabricated MZI device. (b) Enlarged SEM image of the phase shifter integrated on one arm of the MZI. (c) Cross-sectional SEM image of the PCM phase-shifter active region. (d) Schematic top view of the overall device layout. (e) Schematic cross-section of the phase-shifter active region.

## Results

### Electrical Characterization of ITO Microheaters

For all experiments and device measurements, two ITO filaments were characterized in parallel. The voltage–current characteristics of microheaters with varying length were recorded, as shown in Figure 2a, to evaluate their resistance stability and maximum power-handling capability. Notably, linearity was observed near zero bias, indicating the absence of Schottky barriers and confirming the integrity of the ITO–metal interfaces.^31^ For all heater lengths, device failure occurred at a visually detectable rate once the total current reached 30 mA under DC bias. The *V*–*I* characteristics of the ITO filaments exhibit a gradual increase in resistance with applied voltage, reflecting Joule heating. To extract the intrinsic resistance at room temperature, we employed a transmission line method measurement using a low bias of 0.1 V per 20 μm, yielding 2.37 Ω/μm and 23.8 Ω per contact pad (Figure 2b).^32^ The resistivity is lower than the four-probe estimate due to conductivity enhancement from annealing.^33^ To investigate the dependence of filament resistivity on electrical stress, we applied pulses of varying width and amplitude to filaments of different lengths, recording the resulting current response. The measurement setup is shown in Support Information Section S2. From these measurements, a map of the normalized resistance as a function of pulse width and peak electrical power was constructed (Figure 2c), providing insight into the Joule-heating-induced resistance changes. The resistivity exhibits a clear monotonic increase with peak electrical power in the two-filament heater system, consistent with a positive temperature coefficient of resistance. At elevated temperatures, enhanced electron–phonon scattering reduces carrier mobility, leading to increased resistivity.^34^ At a fixed power density, the failure threshold strongly depends on the pulse width, as longer pulses impose a larger cumulative thermal load on the heaters within a given time window.

**Figure 2:**
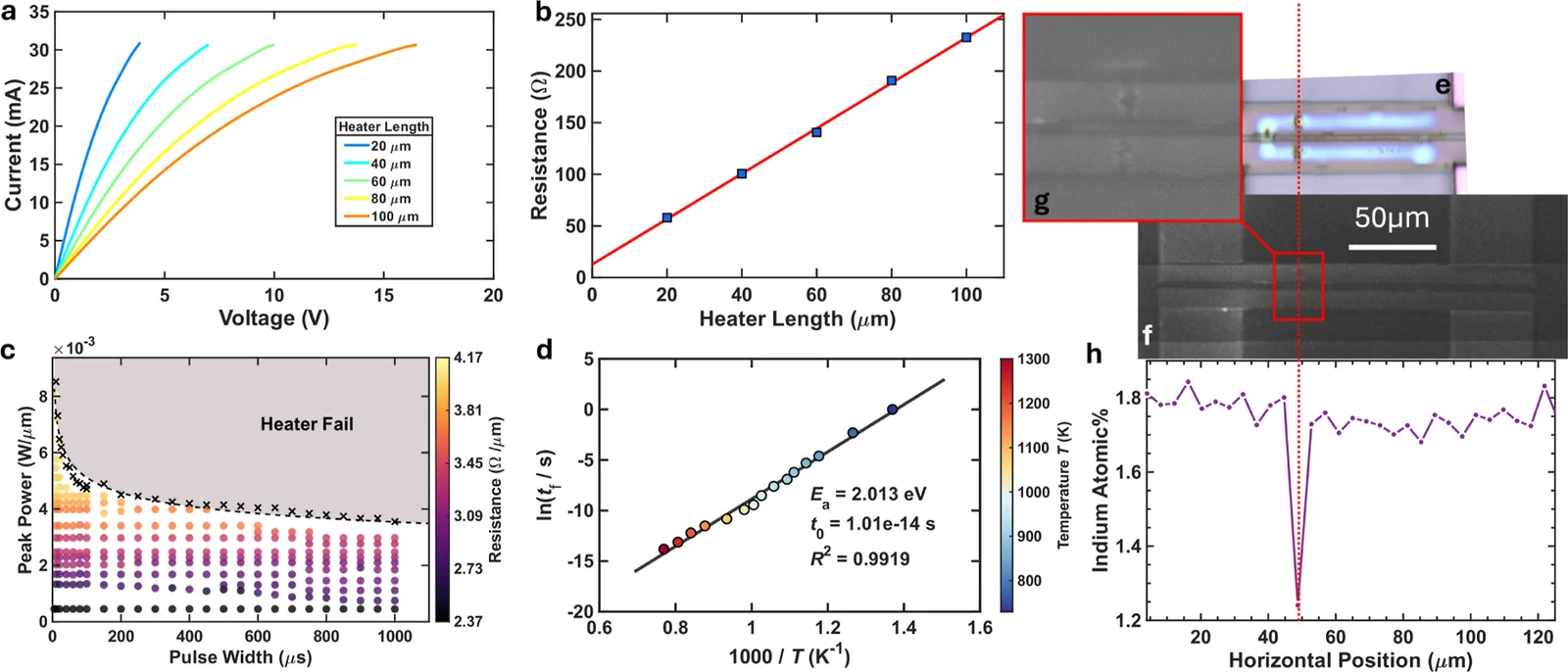
Current–voltage characteristics of ITO filaments with varying lengths (a) Current–voltage characteristics of ITO filaments with varying lengths. (b) Ohmic resistance of ITO filaments with varying lengths (measured at 0.1 V per 20 μm) (c) Normalized resistance of a pair of ITO filaments versus pulse cycles for different pulse widths and corresponding pulse peak powers. (d) Arrhenius fitting of the time–temperature superposition for ITO failure. (e) Optical microscopy image of the fractured ITO filament. (f) Top-view SEM image of the PCM phase shifter containing the broken ITO filament. (g) Magnified SEM image of the fracture region of the ITO filament. (h) EDS line-scan analysis across the ITO filament. The *x*-axis represents the scan position along the filament from left to right (as indicated above), while the *y*-axis corresponds to the detected indium atomic density.

We further analyzed the voltage-resistance data located at the conductive filament failure boundary. Measurements were performed under direct current conditions as well as under pulsed operation with a minimum pulse width of 1 μs. In addition, multiphysics simulations were carried out using *COMSOL Multiphysics* to extract the corresponding failure temperature of ITO (detail about the model is in Support Information Section S3). The obtained data were subsequently fitted using the Arrhenius analysis model

1 $$\ln t=\ln t_{0}+\frac{E_{a}}{k_{B}}\cdot \frac{1}{T}$$

The fitting results are presented in Figure 2d. *k*_B_ denotes the Boltzmann constant, which links energy and temperature. The Arrhenius fit yields an activation energy of *E*_a_ = 2.01 eV and a pre-exponential factor of *t*_0_ = 1.01 × 10^–14^ s, with an excellent coefficient of determination *R* ^2^ = 0.9919. In statistical terms, *R* ^2^ quantifies the proportion of the variance in the observed data that is captured by the fitted model. The activation energy of approximately 2.01 eV is consistent with the energy scale of thermally activated point defect formation,^35,36^ suggesting the failure is governed by thermally driven lattice rearrangement. Furthermore, the extracted pre-exponential factor is on the order of 10^–14^ s, which is comparable to the characteristic time scale of atomic vibrational processes (∼10–14 – 10^–13^ s),^37^ confirming the physical consistency of the fitted kinetic parameters.

Figure 2e shows optical micrographs of failed heaters, where pronounced breakdown along each filament is clearly observed. Energy-dispersive X-ray spectroscopy (EDS) performed at the fractured regions reveals a significantly reduced indium atomic density compared with intact sections of the heaters (in Figure 2h). Under high-bias conditions, the microheaters reach their operational limit as excessive temperature. The resulting atomic displacement promotes the formation of localized conductive paths, causing severe current crowding and runaway thermal loading, ultimately leading to catastrophic breakdown of the ITO layer.^38,39^

### Operational Boundaries

Based on the preceding characterization of the ITO heaters, the filaments are expected to fail beyond a critical electrical stress. Accordingly, we investigate the phase shift as a function of peak electrical power during the initial cycling, using an 80 μm-long PCM phase shifter integrated in a MZI. Support Information Sections S4–S6 show that both C-band and O-band devices exhibit a consistent linear dependence of phase shift on PCM length. The operational envelope of the device was mapped by systematically varying the reset-pulse (amorphization) power and pulse width (under 100 μs) to identify the limits of nonvolatile phase modulation. For each data point, the same set (crystallization) peak power and width of the pulse (0.0035 W/μm, 600 μs) were applied to ensure consistent conditions. At low peak power of reset-pulse, no detectable phase transition was observed. The minimum power density required to induce a phase change increases as the pulse width decreases, reflecting insufficient thermal accumulation under shorter pulses. This lower boundary of the phase-transition regime is indicated by the upper boundary of the no-shift region in Figure 3a. Conversely, at excessively high peak power of reset-pulses, the ITO heater undergoes rapid electrical failure due to resistive breakdown, defining the lower boundary of the failure region. Together, these two boundaries delineate the effective reset window of the device. Notably, although both boundaries exhibit similar trends with pulse width, the operational window narrows dramatically as the pulse width approaches 5 μs and eventually collapses. In this regime, irreversible ITO heater failure occurs before sufficient thermal energy can be delivered to induce phase shift, thereby precluding any viable reset operation. Within the accessible working window, larger pulse power at a fixed pulse width results in increased phase shift, reaching a maximum modulation efficiency of 0.018 rad/μm.

**Figure 3:**
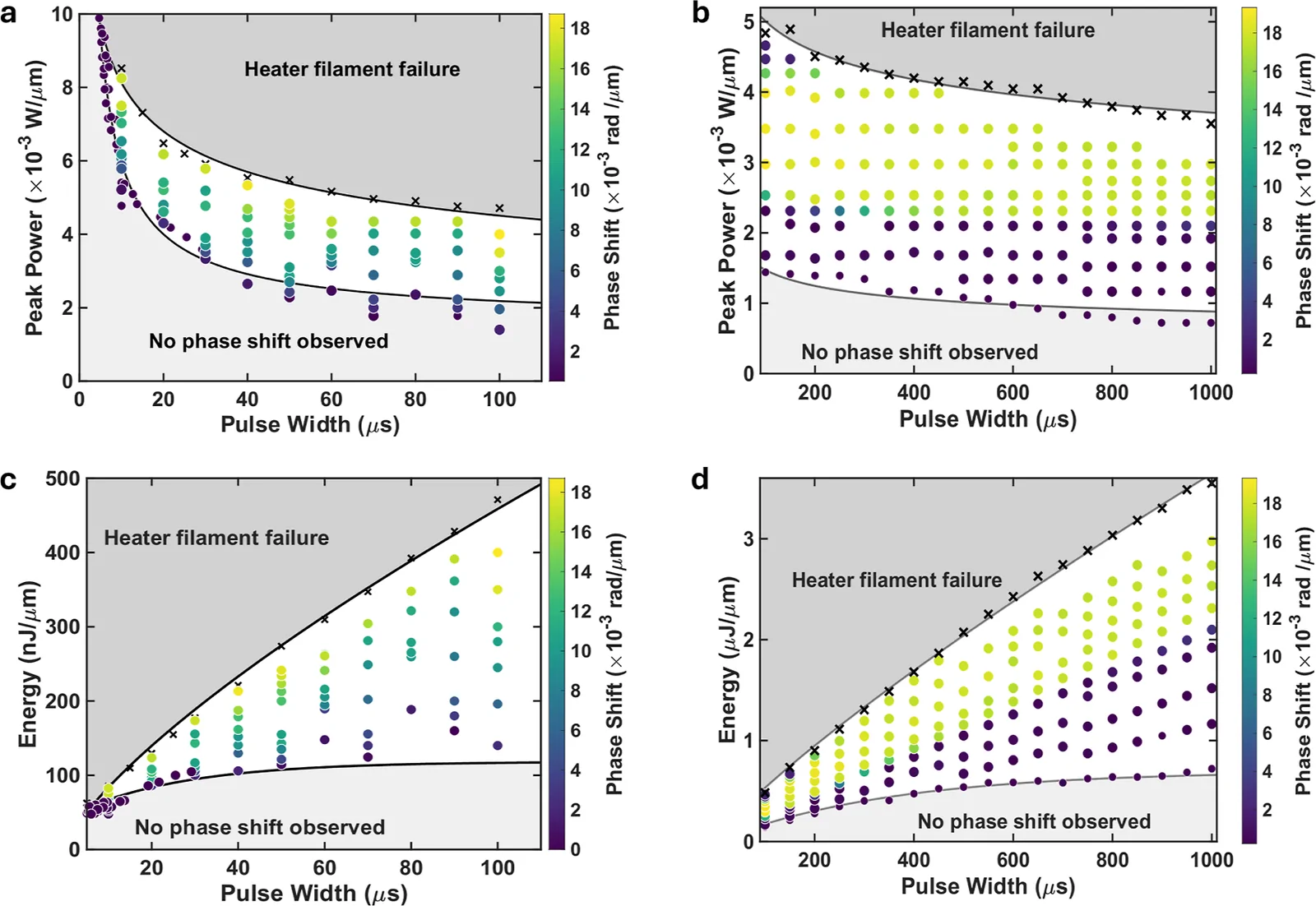
Operational switching window of a normalized length PCM switch. (a) Operational switching window of the ITO heaters for reset. (b) Operational switching window of the ITO heaters for set. (c) Switching energy trend for reset. (d) Switching energy trend for set.

Furthermore, the operational boundaries of the set process were investigated, as shown in Figure 3b. Increasing the pulse duration beyond 100 μs requires lower electrical power to reach the set temperature of the PCM, due to enhanced thermal dissipation over longer time scales. For each data point, a fixed reset pulse (20 μs, 0.006 W/μm) was applied to return the device to a reference state. A stable working window is observed for the set operation. Compared with the reset process, the boundary evolves more gradually with pulse width and does not exhibit closure within the explored parameter range. For pulse widths exceeding 250 μs, all pulses below the failure threshold consistently achieve full set. In contrast, for pulse widths shorter than 200 μs, increasing the power density does not significantly improve the set response, which is attributed to partial crystallization induced by short, high-power pulses. These observations indicate a trade-off between pulse width, energy consumption, and reliable switching. Energy analysis (Figure 3c,d) shows that shorter pulses reduce the total energy required for a given phase shift, but impose stricter constraints on reliable operation.

Based on these results, long-term reliability experiments were performed using a reset pulse of 20 μs at 0.006 W/μm and a set pulse of 600 μs at 0.0035 W/μm, ensuring operation within the stable working window while maintaining low energy consumption and repeatable phase modulation.

### Endurance

In this section, a C-band MZI with 30 μm PCM phase shifters is used to assess long-term reliability. The device exhibits a total insertion loss of approximately 1.1 dB. In this work, cycling endurance is defined as the ability of the PCM device to repeatedly switch between two distinguishable optical transmission states under compensated programming conditions. However, as the phase shift can be scaled with the length of PCM. so for this 30 μm device we define the failure as unswitchable when extionction ratio continuing decreasing under an increasing applied voltage.

During the nonvolatile switching cycles, the reset pulse is 0.006 W/μm with a duration of 20 μs, and the set pulse is 0.0035 W/μm with a duration of 600 μs. Due to the limitation of maximum output voltage stated in Support Information Section S4. An MZI with 30 μm-long Sb_2_Se_3_ phase shifters was subjected to continuing reset and set cycles, during which the corresponding transmission shift were monitored in real time. Figure 4 illustrates the normalized phase shift efficiency and the transmission spectrals along the whole switching process, and the extracted effective refractive index change during repeated cycling. At the initial cycling stage, the device exhibited a switching extinction ratio (ER) of approximately 22 dB, corresponding to a phase-shift efficiency of approximately 0.02, rad/μm. The phase-shift efficiency decreased during the initial cycling period, falling to approximately 0.01–0.004, rad/μm after around 1 × 10^6^ cycles. With continued cycling, the phase-shift efficiency gradually stabilized at approximately 0.004, rad/μm, corresponding to an ER at approximately 6 dB. This initial stabilization process is attributed to interfacial stress and material fatigue within the SiO_2_ cladding.^40–42^ The resistance of the ITO heater exhibits a significant increase after approximately 40 million switching cycles. To maintain the required electrical peak power (0.006 W/μm for reset, 0.0035 W/μm for set) delivered to the heater, the voltage of both the reset and set pulses is increased accordingly (see Support Information Section S7), ensuring a consistent level of phase shift. The phase shift efficiency under a fixed voltage is shown in Support Information Section S8. Under adaptive voltage compensation, the Sb_2_Se_3_-based phase shifters exhibit robust and repeatable operation for up to 140 million switching cycles. Beyond 140 million cycles, the phase change progressively decreased toward zero at a rate of 0.001 rad/μm per 5 million cycles, indicating device failure associated with electrical breakdown of the ITO heaters.

**Figure 4:**
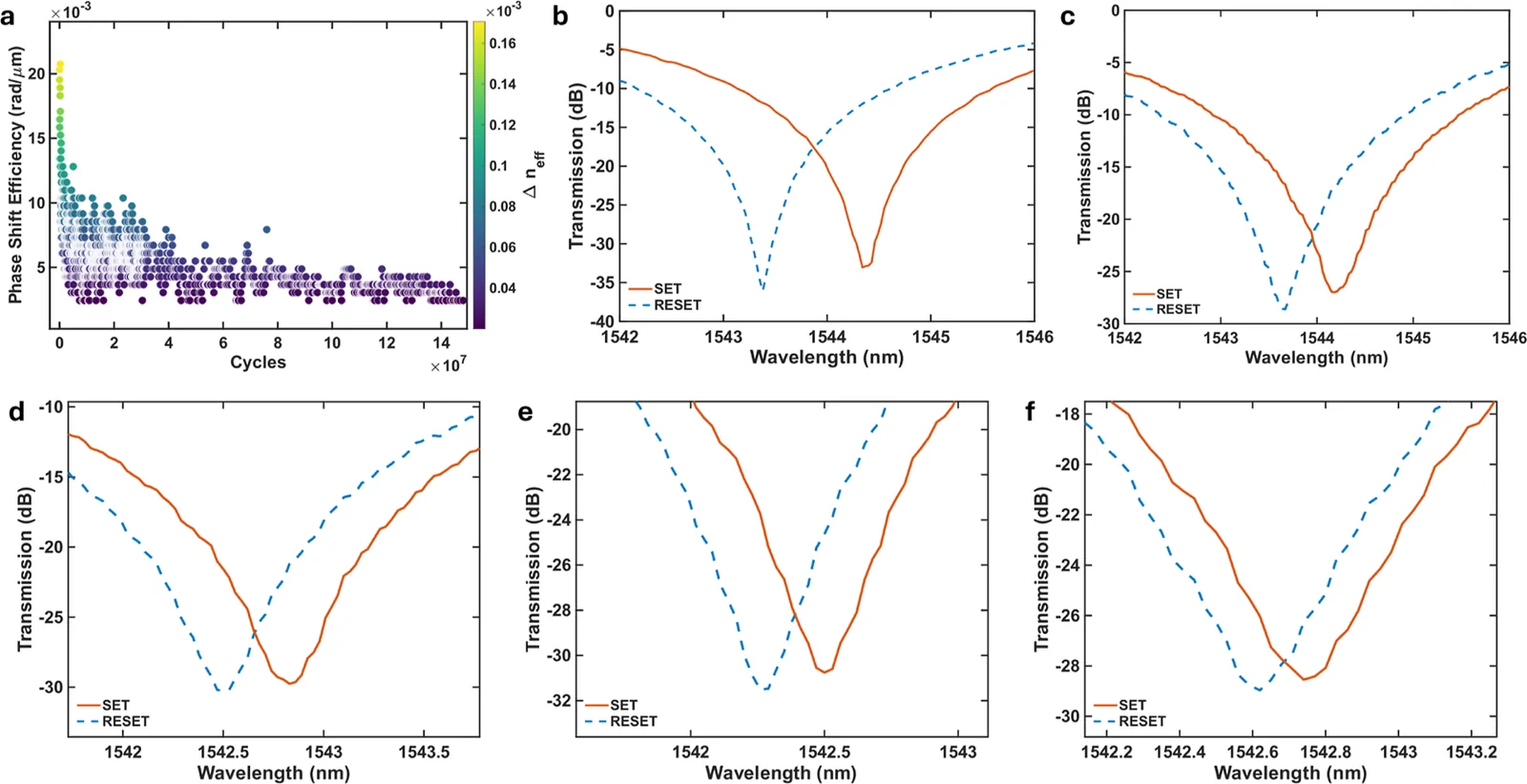
(a) Endurance cycling test results showing the phase shift over 140 million cycles. (b) Transmission spectral of SET/RESET at initial cycle. (c) Transmission spectral of SET/RESET at 1 million cycles. (d) Transmission spectral of SET/RESET at 40 million cycles. (e) Transmission spectral of SET/RESET at 80 million cycles. (f) Transmission spectral of SET/RESET at 140 million cycles.

In Table 1, we summarize representative studies on nonvolatile photonic phase shifters. Our results demonstrate the endurance and bit precision that significantly exceed those of previous reports based on PCM technologies. Moreover, this work substantially narrows the performance gap with state-of-the-art volatile technologies used for digital-analog hybrid matrix multiplication in image processing and deep learning applications.^43,44^ This improvement can be attributed to the careful control of the heater operating boundaries, which ensures stable and reliable device operation over extended cycling. Compared with state-of-the-art low-loss Sb_2_Se_3_ photonic phase shifters, which report a large intrinsic refractive index contrast between the fully crystalline and fully reset phases at telecommunication wavelengths,^16,45,46^ the effective refractive index change and phase shift efficiency we achieved is noticeably smaller. This indicates that the electrically programmed reset state corresponds to an incompletely amorphized configuration rather than a fully amorphous phase.

**Table 1:** Comparison of Non-volatile Photonic Memory Demonstrations

ref	Technology/Material	1 bit endurance	Multibit number and cycles	1 bit Switching Time	1 bit Switching Energy	Shifting Efficiency (rad μm^–1^)	ER (dB)
^47^	MEMS	10^6^	N/A	2.2 μs/7.4 μs	1 pJ	N/A	44.4
 ^48^	Ferroelectric	300	3bit, 300	1 μs	4.6 pJ	0.003	12
 ^49^	Memristor (GaAs-Al_2_O_3_–Si)	1000	1.5 bit, 1000	0.3 ns/0.9 ns	150 fJ/360 fJ	0.0282	14
 ^50^	Memristor PZT	>100000	2.32 bit, 20	2.5 ns	12.3 pJ	0.003	19
 ^51^	Magneto-optic Ce/YIG	2.4 × 10^9^	3.5 bit, 1	1 ns	143 fJ	0.0024	16.2
 ^14^	Sb_2_Se_3_, Graphene heater	1500	3.5bit, 1	408 ns/220 μs	5.55 nJ/753 nJ	0.00035	3
 ^52^	GST, Doped Si	100	3.5bit, 3	50 ns/200 ns	8.8 nJ/6.9 nJ	N/A	4.13
 ^53^	Sb_2_Se_3_, Doped Si	10,000	6bit, 1	135 ns/20 μs	105 nJ/1.2 μJ	0.215	20
 ^54^	GSSe, W/Ti	500,000	4 bit,1	2 μs/20 μs	400 nJ	N/A	6
 ^22^	Sn-GST, ITO	30,000	3.87 bit,16	5 μs/50 μs	N/A	N/A	50
 ^55^	GST, ITO	3	6 bit,1	1.1 μs/60 μs	81 nJ/1.96 μJ	0.04	30
 ^56^	Sb_2_Se_3_, Doped Si	7900	NA	1.5 μs/300 μs	0.54 μJ/19.2 μJ	0.067	12
 ^57^	Sb_2_S_3_, Doped Si	1600	5 bit	150 ns/200 ms	197 nJ/12 mJ	0.1	10
This work	Sb_2_Se_3_, ITO	1.4 × 10^8^	6 bit, 10	20 μs/600 μs	3.6 μJ/63 μJ	0.004	29

### Multilevel Modulation

To explore the achievable bit precision in PCM-based MZIs, a device incorporating an 80 μm-long PCM section in one arm was characterized. The device exhibits a total insertion loss of approximately 2 dB. A reset of 15.4 V, 40 μs pulses was used for amorphization, while 10.4 V, 600 μs pulses were applied for crystallization. The measured transmission spectrum over a single free spectral range (1550–1560 nm) is shown in Figure 5a, comparing the initial, amorphous, and crystalline states. An ER of up to 29 dB is achieved at 1551.75 nm, with a maximum wavelength shift of 2.5 nm between states.

**Figure 5:**
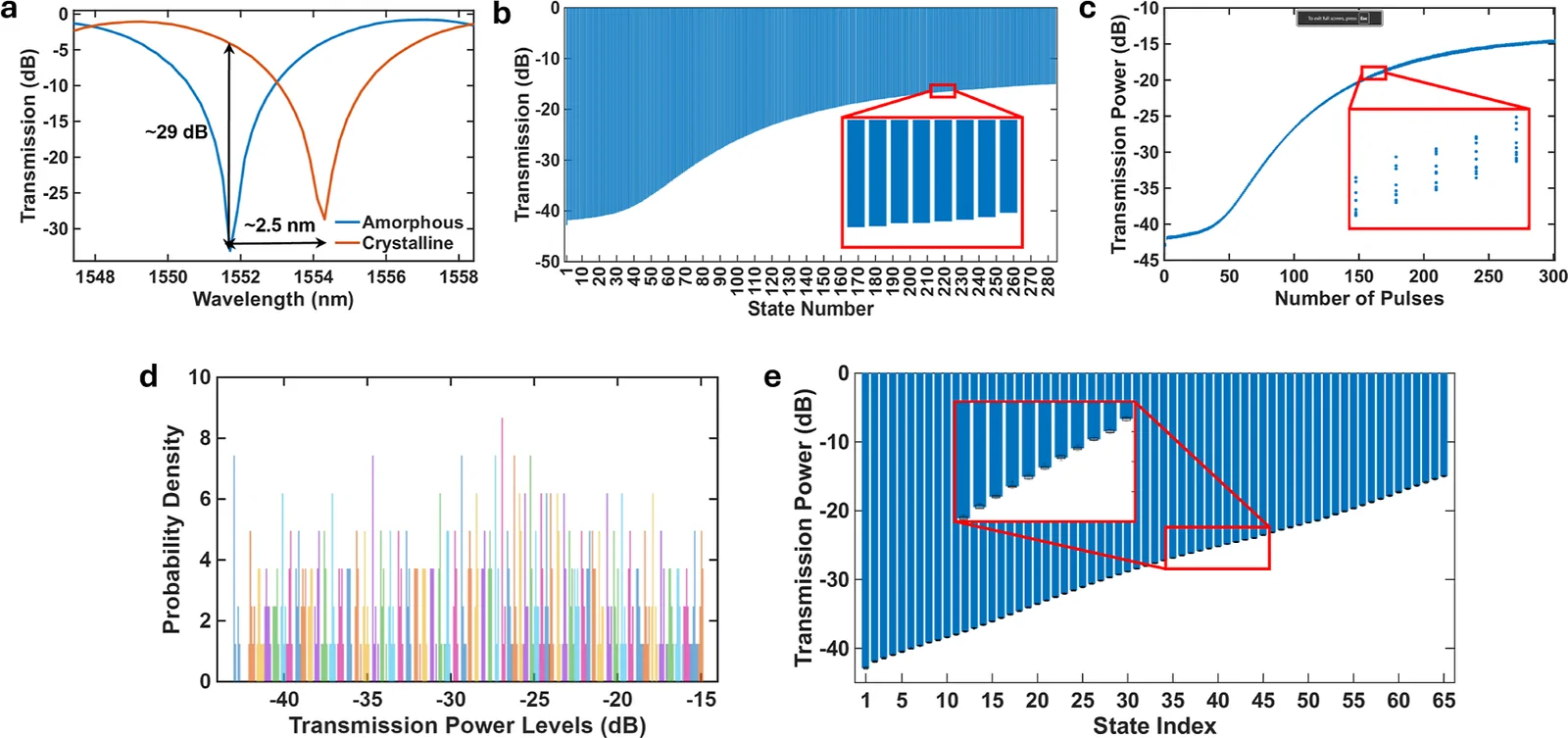
Multilevel modulation based on a 80 μm length PCM switch. (a) Initial measurement for Amorphous and crystalline states’ spectrum. (b) Gradual crystallization of the PCM using continues crystallization pulses with lower width. (c) Multiple repetition of gradual crystallization, capturing the transmission power level. (d) Distribution of power across 65 states, including the representation of the 65 discrete levels with overlaid boxplot analysis. (e) The probability density functions for all discrete states, with standard deviation values varying from 0.0566 to 0.1265.

Multilevel operation was further demonstrated via gradual crystallization. Starting from the amorphous state, a sequence of 300 identical pulses (10.4 V, 80 μs) was applied while monitoring the transmission at 1551.75 nm. The transmission increases monotonically with pulse number, indicating progressive phase transitions in the PCM. As shown in Figure 5b, this process yields 284 distinguishable transmission levels spanning an aggregate dynamic range of 27 dB, corresponding to an average step size of 0.095 dB between adjacent levels.

Repeatability was evaluated over ten independent switching cycles. In each cycle, the PCM was reinitialized to the amorphous state using 15.4 V, 40 μs pulses, followed by the same crystallization sequence. The resulting transmission traces, presented in Figure 5c, exhibit strong overlap, confirming stable and repeatable operation across cycles.

To quantify the statistical distribution of the transmission states, each level is modeled as a Gaussian distribution with mean μ_*n*_ and standard deviation σ_*n*_. The probability density function of the *n*-th state is given by

2 $${PDF}_{n}=\frac{1}{\sigma_{n}\sqrt{2\pi }}\exp\left[-\frac{{\left(P_{n}-\mu_{n}\right)}^{2}}{2\sigma_{n}^{2}}\right]$$

Based on the overlap of adjacent distributions, the number of reliably distinguishable states is determined to be 65, corresponding to a symbol error rate (SER) of 1.2 × 10^–2^. The distributions of all states are summarized in Figure 5d,e with standard deviations ranging from 0.0566 to 0.1265 dB, confirming the robustness of multilevel operation.

### High-Baud-Rate Data Transmission

To evaluate the compatibility of the proposed photonic switch with high-speed optical transmission, we carried out transmission experiments using on–off keying (OOK), 4-level pulse amplitude modulation (PAM4), and 8-level pulse amplitude modulation (PAM8). Although the PCM switching process itself is not intended to operate at the data modulation rate, the nonvolatile PCM device can maintain a programmed optical state while carrying high-speed data, making it a potentially useful element for reconfigurable optical switching networks. The experimental setup for the high-speed transmission measurements is illustrated in Support Information Section S8. The device used in this section incorporates a 120 μm-long PCM section, which enables a full π phase shift. The device exhibits a total insertion loss of approximately 2.5 dB. The wavelengths corresponding to the maximum transmission are 1545.32 and 1540.56 nm for the set and reset states, respectively. The initial measured transmissions are shown in Figure 6.

**Figure 6:**
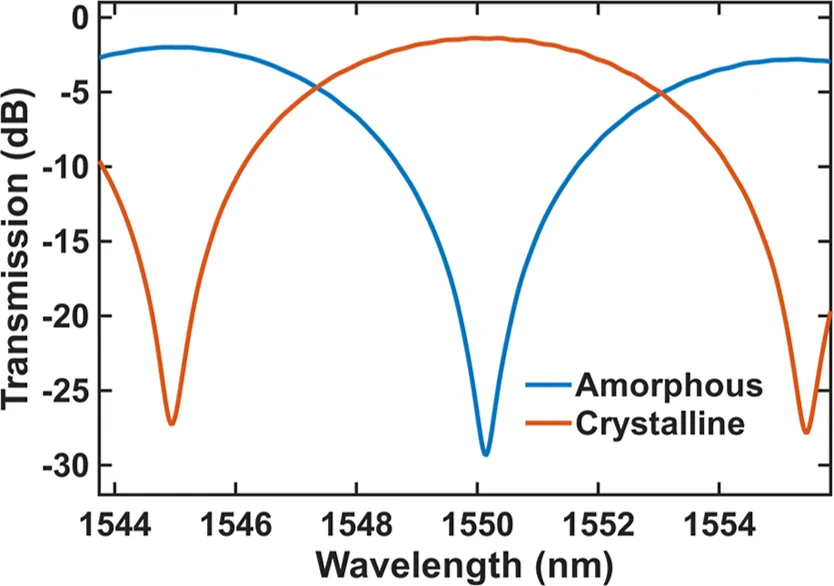
Initial measurement for Amorphous and crystalline states’ spectrum of an MZI with 120 μm-long PCM sections.

Figure 7 presents the measured BER as a function of received optical power (ROP) for the 256-GBd OOK, 175-GBd PAM4, and 90-GBd PAM8 signals. The B2B reference was measured at 1545.32 nm, while transmission measurements for the amorphous and crystalline PCM states were performed at their respective MZI transmission peaks of 1545.32 and 1540.56 nm. Across all modulation formats, both PCM states exhibit comparable BER performance, with the crystalline state showing slightly lower BER at high ROP.

**Figure 7:**
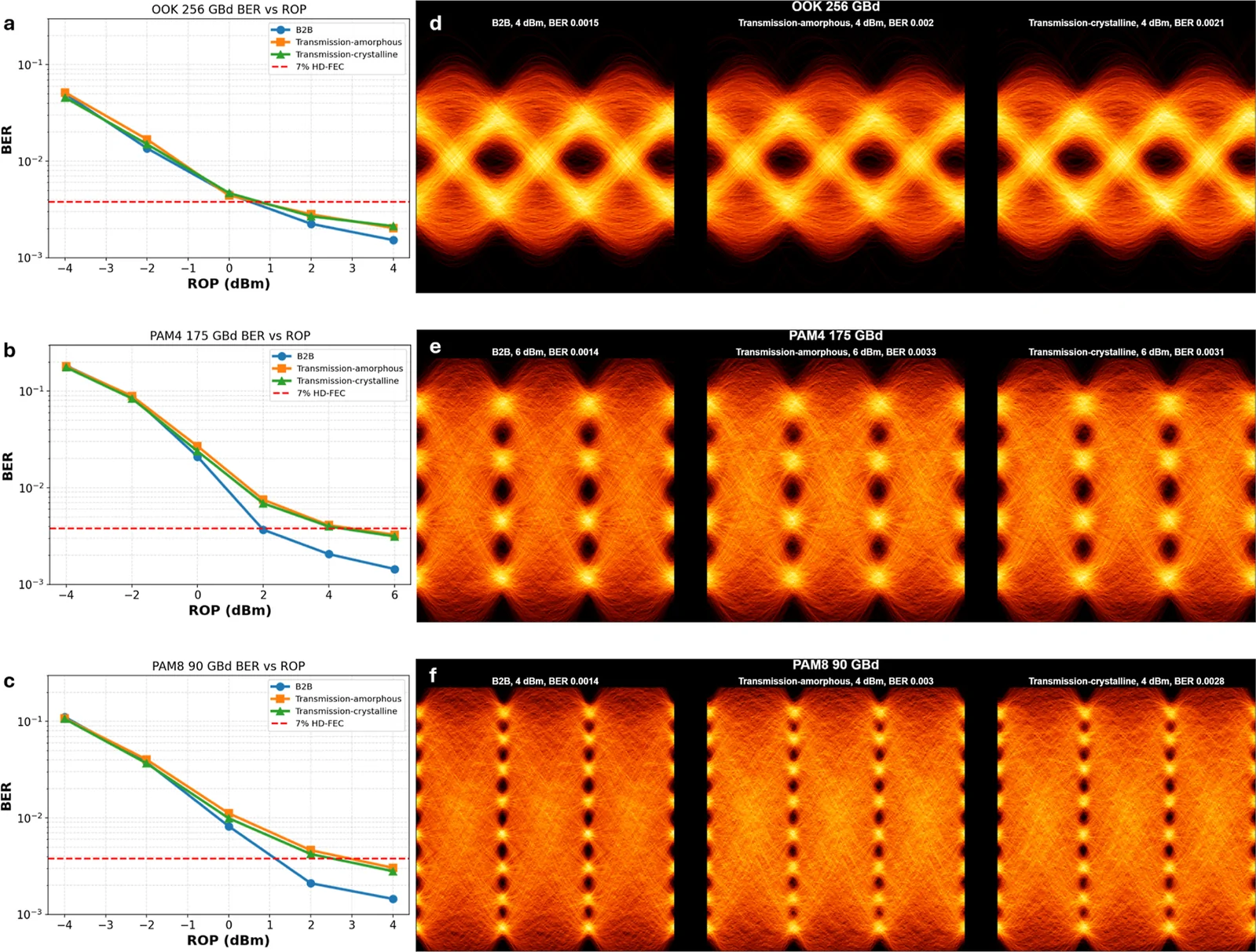
High-speed transmission performance of OOK, PAM4, and PAM8 signals through a PCM-loaded MZI. (a-c) Measured BER versus received optical power (ROP) for 256-GBd OOK, 175-GBd PAM4, and 90-GBd PAM8 signals under back-to-back (B2B) conditions and after transmission through the PCM-loaded MZI in its amorphous and crystalline states. The B2B reference and amorphous-state transmission were measured at 1545.32 nm, while the crystalline-state transmission was measured at 1540.56 nm, corresponding to the respective MZI transmission peaks. The dashed line indicates the 7% hard-decision forward-error-correction (HD-FEC) threshold of 3.8 × 10^–3^. (d-f) Reconstructed post-DFE eye diagrams for 256-GBd OOK, 175-GBd PAM4, and 90-GBd PAM8 signals under B2B conditions and after transmission through the PCM-loaded MZI in its amorphous and crystalline states. The B2B reference and amorphous-state transmission were measured at 1545.32 nm, while the crystalline-state transmission was measured at 1540.56 nm, corresponding to the respective MZI transmission peaks.

Both PCM states achieve BER values below the 7% HD-FEC threshold of 3.8 × 10^–3^. The 256-GBd OOK signal crosses this threshold between 0 and 2 dBm ROP, whereas the 175-GBd PAM4 and 90-GBd PAM8 signals cross between 4 and 6 dBm and 2–4 dBm, respectively. Compared with the B2B reference, OOK exhibits a relatively small transmission penalty, while more pronounced penalties are observed for PAM4 and PAM8.

The reconstructed post-DFE eye diagrams show clearly distinguishable levels for all modulation formats and PCM states. The amorphous and crystalline states exhibit similar level separation, consistent with their comparable BER performance. As the PAM order increases, reduced spacing between adjacent levels leads to smaller eye openings and increased sensitivity to transmission-induced noise and distortion.

## Conclusion

We demonstrate a scalable, electrically driven, multilevel photonic computing platform based on a nonvolatile, electrically reconfigurable phase shifter employing the low-loss phase-change material Sb_2_Se_3_ integrated with ITO microheaters on an 8-in. Si_3_N_4_ fabrication platform. Wafer-level mapping of the phase-shift efficiency further demonstrates the consistency of the integrated devices across the wafer (see Supporting Information, Section S5).

The device sustains approximately 1.4 × 10^8^ reversible 1 bit switching operations, achieving a phase-shifting efficiency of 0.004 rad/μm and an effective refractive index contrast of Δ*n*_eff_ ≈ 0.003, as measured experimentally. This operating point (0.004 rad/μm) corresponds to conditions well within the stable switching window of Figure 3a, prioritising endurance over maximum phase-shift amplitude.

The reported endurance is derived from full set/reset under accelerated elevated-temperature conditions, representing a worst-case operating environment. The Arrhenius analysis was performed independently to evaluate the temperature dependence of the ITO heater failure mechanism and was not used to derive the experimentally demonstrated cycling endurance. In practical applications, devices predominantly undergo nondestructive read operations at low optical power, interspersed with relatively infrequent write events. Consequently, the effective operational lifetime under realistic workloads is expected to exceed that under continuous full set/reset cycling. For application scenarios such as optical circuit switching (OCS) in data centers, programmable photonic FPGAs, and synaptic weight updates in optical neural networks (ONNs), the typical switching demand is on the order of 10^4^–10^5^ write operations per day. This corresponds to a projected device lifetime exceeding approximately 4 years, and substantially longer under read-dominated operation, highlighting the suitability of the device for long-term system-level deployment.

The measurements indicate that device endurance is presently limited by heater failure rather than intrinsic PCM degradation. Replacing the ITO heater with more thermally robust transparent conductors (e.g., IZO, AZO, or Nb-doped TiO_2_) may therefore decouple the endurance ceiling from the PCM and potentially extend the switching lifetime beyond 10^9^ cycles. In addition, such materials could sustain higher peak power, enabling faster thermal transients and enhanced quench rates, which may improve amorphization efficiency and yield larger phase-shift contrast.^58,59^

Multilevel operation enabled by continuous pulse modulation allows continuous and deterministic tuning of the optical transmission. In our device, 284 optically resolvable levels collapse to 65 repeatable levels, corresponding to a measured state error rate (SER) of 1.2 × 10^–2^. The observed limitation in the number of repeatable states may be related to stochastic processes during partial crystallization of the PCM. Within the Johnson–Mehl–Avrami–Kolmogorov (JMAK) framework, stochastic nucleation during partial crystallization could lead to variations in the crystalline fraction and, consequently, in the effective refractive index for nominally identical programming pulses. Other factors, including heater-resistance evolution, pulse-amplitude fluctuations, thermal nonuniformity, optical wavelength drift, and variations in the MZI transfer function, may also contribute to the observed state-to-state variability.

Further reduction of SER can be achieved by increasing the phase-change efficiency to produce larger effective index shifts per level, thereby improving level separation, as well as through optimization of the electrical driving scheme. Currently, multilevel writing is performed in an open-loop fashion, applying a fixed pulse sequence without real-time feedback. While this allows high-speed programming, it is inherently limited by stochastic nucleation. Incorporating a closed-loop feedback scheme, where the optical output is monitored during writing and used to adjust subsequent pulses (optical servo), would improve multilevel precision at the cost of increased write latency. This trade-off between speed and fidelity positions our work within the context of emerging PCM photonic memory architectures and provides a clear pathway for future optimization of device performance.

Alternatively, the phase shift can be increased by extending the length of the PCM section. A full (π) phase shift is achieved when the length of the PCM-based phase shifter is increased to (120 μm). Moreover, all the proposed MZIs exhibit an ER exceeding (25 dB). However, increasing the PCM length also enlarges thermal nonuniformity and the stochastic nucleation volume, leading to enhanced shot-to-shot variability. This trade-off implies the existence of an optimal PCM length that maximizes the number of reliable levels for a given SER. If this optimal length is shorter than that required for a full phase-amplitude interference shift, a cascaded (multistage) PCM architecture provides a viable route to simultaneously achieve high optical resolution and low SER.

We evaluate the energy efficiency of our multilevel PCM photonic switch by computing the energy per programmed optical state. For our current 6 bit operation (65 repeatable levels), the reset (amorphization) pulse requires approximately 19.2 μJ, while each intermediate set (crystallization) pulse consumes 22.4 μJ per level. This corresponds to an energy-*per*-level figure of merit, allowing direct comparison to conventional electronic memories: Dynamic random-access memory (DRAM) operates at ∼10⁡fJ/bit , and Flash at ∼10⁡pJ/bit , highlighting the orders-of-magnitude higher energy cost typical of PCM photonic switches. To further improve energy efficiency, multilevel set pulses can employ the same amplitude as the reset pulses while reducing the pulse width to the ∼10⁡μs scale. This approach delivers the same peak temperature necessary for crystallization, enabling the desired phase-change effect with approximately 100-times lower energy consumption per pulse. This approach situates the device within the broader memory landscape and provides a framework for systematic energy-performance comparisons between PCM photonic devices and conventional electronic memories.

The high-speed transmission experiments further demonstrate the compatibility of the PCM-loaded MZI with advanced optical communication systems. The device successfully supports 256-GBd OOK, 175-GBd PAM4, and 90-GBd PAM8 transmission, achieving BER performance below the HD-FEC threshold in both amorphous and crystalline states. These results highlight the potential of phase-change-material-based photonic switches for reconfigurable, high-speed optical interconnects.

The results in this work provide a comprehensive framework for the development of energy-efficient, broadband nonvolatile photonic systems, paving the way for next-generation optical interconnects and hardware-efficient neural networks.

## Supplementary Material



## Notes

Additional fabrication details, COMSOL thermal simulations, supplementary optical transmission measurements, endurance and experiment setups are available in the Supporting Information (PDF)
