# Supplementary material for: High-Endurance, Low-Loss Sb2Se3 Optical Switches on Silicon Nitride Using Transparent Conductive Heaters
Source: ACS Photonics. 2026 Sep 25;13(19):5894–904. doi: 10.1021/acsphotonics.6c01756 (PMC13651611; doi:10.1021/acsphotonics.6c01756)
Supplement: Supplementary file 1 [file ph6c01756_si_001.pdf]

# Support information: High-Endurance, Low-loss $Sb_2Se_3$ Optical Switches on Silicon Nitride using Transparent Conductive Heaters

Xingshi Yu*[1], Ipsita Chakraborty[1], Isaac Johnson[1], Savvas I. Raptis[2], Qianbin Luo[1], Hao Liu[1], Thalia D. Bucio[1], Elliot Sandell[1], Christos Vagionas[2], Amalia Miliou[2], Periklis Petropoulos[1], Nikos Pleros[2], Ioannis Zeimpekis[3], and Frederic Gardes*[1]

[1]Optoelectronics Research Centre (ORC), University of Southampton , University Road, Southampton, SO17 1BJ, Hampshire, United Kingdom
[2]Wireless and Photonic Systems and Networks (WinPhoS) research group, The Aristotle University of Thessaloniki, 3is Septemvriou Street, Agios Dimitrios, Thessaloniki, 54124, Greece.
[3]Electronics and Computer Science (ECS), University of Southampton , University Road, Southampton, SO17 1BJ, Hampshire, United Kingdom.

*Email: Xingshi.Yu@soton.ac.uk, fg5v@ecs.soton.ac.uk

## Experimental Methods

## 1 Fabrication

The fabrication process begins with the Low Pressure Chemical Vapor Deposition (LPCVD) deposition of a 400 nm thick $Si_3N_4$ layer on a silicon substrate with a 3.2 $\mu$m thermal oxide undercladding. Waveguide geometries are patterned using high-resolution 248 nm Deep UV lithography and subsequently defined through reactive ion etching (RIE). Following core definition, a $SiO_2$ cladding is deposited and planarized using chemical mechanical polishing (CMP) to achieve the target 20 nm optical spacer thickness above the waveguide surface. The resistive microheaters are then formed using an 80 nm thick indium tin oxide (ITO) film deposited via physical vapor deposition (PVD). An annealing process follows to improve the ITO conductivity[1, 2]. Electrical contact is established through the deposition of Ti/TiN/Al/Ti/Ni (top to bottom) pads. The active 20 nm thick $Sb_2Se_3$ layer is integrated via radio-frequency (RF) magnetron sputtering. To prevent degradation of the PCM during the followup fabrication steps, a 40 nm thick ZnS:$SiO_2$ (20%:80%) protection cladding is deposited immediately following the PCM growth. Finally, a conformal $SiO_2$ oxide cladding layer is deposited over the entire device stack, encapsulating the $Sb_2Se_3$ layer, ITO micro-heaters, and waveguide structures. This oxide overcladding suppresses oxygen adsorption at the ITO surface, thereby mitigating resistivity degradation of the heater [3, 4].

## 2 Electrical characterization of the normalized resistivity of a pair of ITO filaments

A Keithley 2401 SourceMeter was used to apply a DC voltage to a pair of ITO heaters through four electrical probes with needle tips. The probes were connected to the Keithley instrument via Bayonet Neill–Concelman (BNC) cables. A Python script was used to control the SourceMeter and record the current data.

The normalized resistivity of a pair of ITO filaments in the PCM phase shifter was characterized using a pulsed electrical measurement scheme. Figure S1 shows the connection of the instruments. A pulse generator (PG, BNC577) was used to apply a single electrical pulse with peak voltage $U_1$ and pulse width $T_1$.

The output pulse was applied to a series circuit consisting of the PCM phase shifter and a 50-Ω load provided by channel 2 of an arbitrary waveform generator (AWG, Agilent 33600A). The PCM phase shifter contains two parallel ITO filaments acting as the resistive heater.

The voltage generated by the pulse generator ($U_1$) and the voltage across the 50-Ω load ($U_2$) were monitored using channel 1 and channel 2 of the AWG, respectively. Since the input impedance of channel 1 (1 MΩ) is much larger than the resistance of the device and the 50-Ω load, its influence on the circuit can be neglected.

The current flowing through the circuit was determined from the voltage drop across the 50-Ω load according to

$$I_1 = \frac{U_2}{50\,\Omega}. \tag{1}$$

The instantaneous electrical power delivered to the PCM phase shifter was calculated as

$$P_{\text{peak}} = (U_1 - U_2)I_1. \tag{2}$$

The effective resistance of the device was obtained from

$$R_{\text{device}} = \frac{U_1 - U_2}{I_1}, \tag{3}$$

which includes the resistance of the ITO filaments and the contact resistance of the metal pads ($R_{\text{device}} = R_{\text{ITO}} + R_{\text{pad}}$). By subtracting the previously calibrated pad resistance, the normalized resistance of the pair of ITO filaments was extracted as a function of the applied peak power.

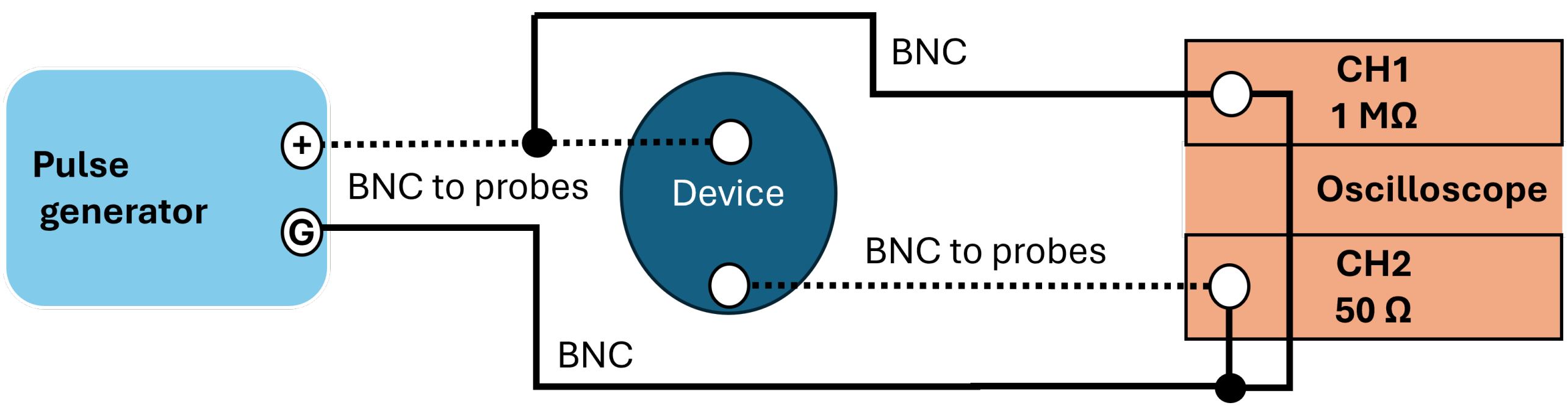


Figure S1: Experiment instruments connection for heater measurement.

## 3 COMSOL Joule-Heating Model

The temperature used for Arrhenius analysis is obtained from a finite-element Joule-heating model implemented in COMSOL Multiphysics. To validate the reliability of the extracted activation energy, an independent experimental calibration of the device temperature is performed using the thermo-optic response of a MZI. The device operates at a wavelength of 1550 nm, with an ITO heater length of 92 $\mu$m.

The COMSOL model incorporates the full device geometry (see main tex Figure 1(e)), with material parameters summarized in Table S1, including electrical conductivity, thermal conductivity and heat capacity for all constituent layers ($Si_3N_4$, ITO, PCM and ZnS:$SiO_2$). A non-uniform tetrahedral mesh is employed, with local refinement in the heater region down to a minimum element size of 10 nm to accurately resolve steep thermal gradients.

Under DC bias below the phase-transition threshold, the measured optical phase shift is assumed to be dominated by the thermo-optic effect. The phase shift $\Delta\phi$ in the MZI is related to the effective refractive index variation $\Delta n_{\text{eff}}$ as

$$\Delta\phi = \frac{2\pi}{\lambda}\Delta n_{\text{eff}} L \tag{4}$$

where $L$ is the interaction length and $\lambda$ is the operating wavelength. The refractive index variation is in turn related to the temperature rise via the thermo-optic coefficient,

$$\Delta n_{\text{eff}} = \frac{dn_{\text{eff}}}{dT}\Delta T \tag{5}$$

where $dn_{\text{eff}}/dT$ denotes the effective thermo-optic coefficient of the waveguide. Here, a value of $2.57 \times 10^{-5}$ K$^{-1}$ is adopted, consistent with reported values for $Si_3N_4$ waveguides at 1550 nm [5]. Combining the above expressions yields

$$\Delta T = \frac{\Delta\phi, \lambda}{2\pi L(dn_{\text{eff}}/dT)} \tag{6}$$

This analysis assumes that the phase response is dominated by the thermo-optic effect in $Si_3N_4$. The extracted temperature therefore represents an effective spatial average along the interferometer arm, while the actual temperature distribution may be non-uniform.

In Figure S2, the experimentally extracted temperature is compared with the simulated temperature obtained under identical electrical bias conditions. The corresponding electrical power predicted by the model is also benchmarked against experimental measurements. Excellent agreement is observed over the full voltage range (0–9 V), with a mean relative error of 0.00337 and a coefficient of determination of $R^2 = 0.9970$, confirming the accuracy of the electrothermal model.

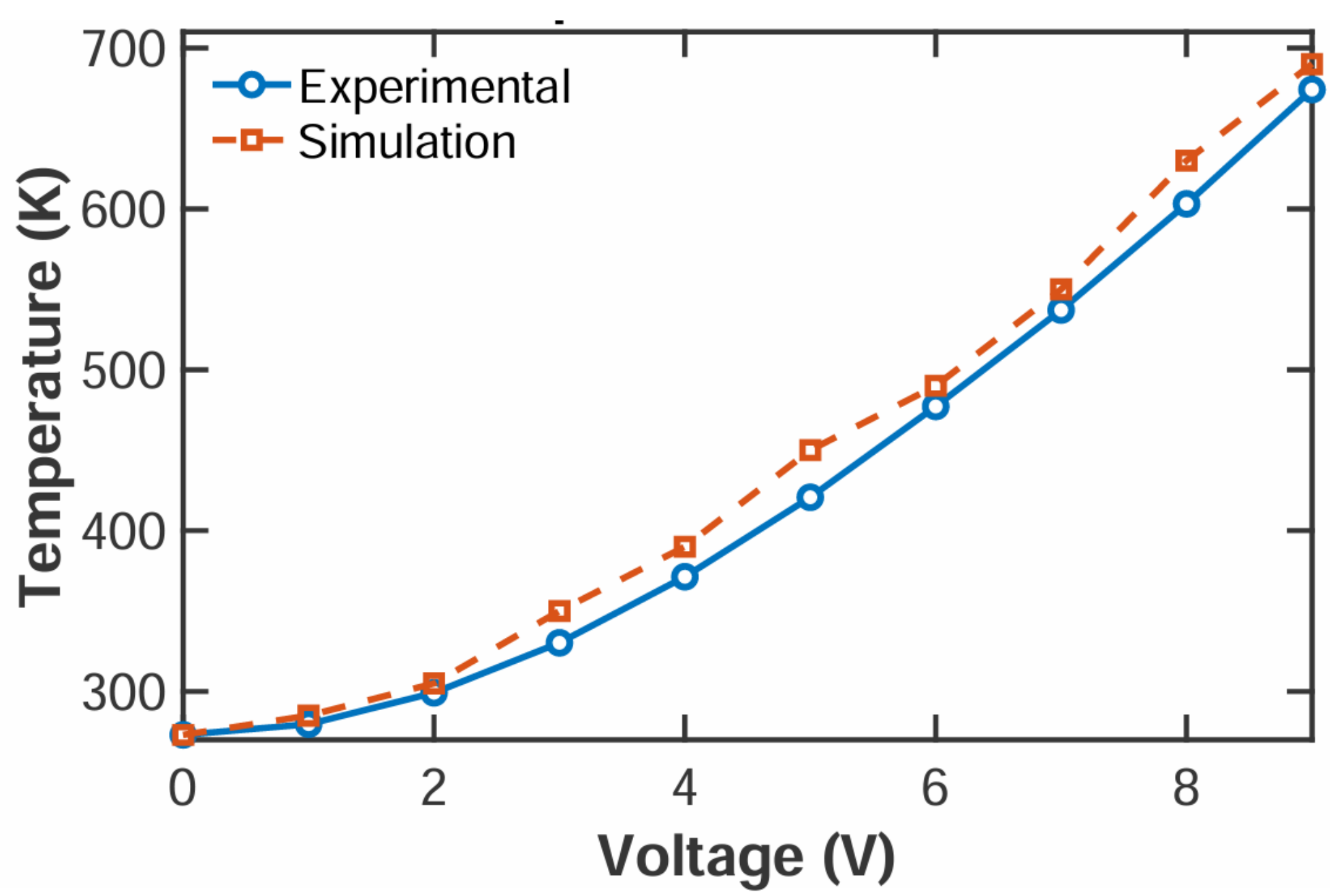


Figure S2: Comparison between simulated temperature and experimentally extracted temperature from optical phase shifts.

# 4 Optoelectronic cycling and transmission measurements

The optoelectronic cycling experiment was performed on a MZI incorporating a PCM phase shifter in each arm. The PCM was initially in the crystalline state. All instruments were controlled using a custom Python program. The instrument connection is shown in Figure S3.

The optical transmission from MZI input 1 to output 1 was characterized using a lightwave multimeter (Agilent 8163B). A tunable laser module (81940A) provided wavelength sweeps across the C-band, while the transmitted optical power was recorded using a calibrated power sensor (81634B).

Table S1: Thermal material properties used in the COMSOL model.

| Material | Thermal Conductivity (W/m·K) | Density ($kg/m^3$) | Heat Capacity $C_p$ (J/kg·K) |
|---|---|---|---|
| $Si_3N_4$[6] | 30 | 3180 | 670 |
| ITO | 4 [7] | 7140 [8] | 340 [8] |
| $Sb_2Se_3$ | 0.72 [9] | 5840 [9] | 270 [10] |
| $ZnS:SiO_2$ [a] | 0.6 [11] | 2600 [12] | 700 [12] |

[a] Estimated using a volumetric weighted average.

To accelerate the cycling experiment, electrical pulses were generated using an arbitrary waveform generator (AWG, Agilent 33600A), which was directly connected to the PCM phase shifter. It is capable of delivering voltage pulses with amplitudes up to 10 V. The rise and fall times of the pulses are approximately 100 ns. The AWG produced a periodic waveform with a period of 6 ms, consisting of a reset pulse (0.006 W/$\mu$m, 20 µs) and a set pulse (0.0035 W/$\mu$m, 600 µs). Since the AWG does not provide an internal pulse counter, a pulse generator (PG, BNC577) was used to trigger and gate the AWG output, enabling accurate control of the total number of electrical pulses applied to the device.

After reaching a predefined number of electrical cycles, the optical characterization sequence was triggered. The AWG first applied an additional reset pulse to amorphize the PCM. After a waiting time of 10 ms to ensure thermal relaxation, the laser performed a wavelength sweep and the optical transmission spectrum was recorded. Subsequently, a set pulse was applied to recrystallize the PCM, followed by a second optical transmission measurement. The phase shift between these two switching events can be extracted from the spectral shift between the corresponding transmission minima, denoted as $\Delta\lambda$:

$$\Delta\lambda = \lambda_{\text{set}} - \lambda_{\text{reset}}, \tag{7}$$

The phase shift $\Delta\phi$ can be expressed as

$$\Delta\phi = 2\pi \frac{\Delta\lambda}{\text{FSR}}, \tag{8}$$

where FSR is the free spectral range of the interferometer.

Accordingly, the phase-shifting efficiency $\eta$ is defined as

$$\eta = \frac{\Delta\phi}{L} = \frac{2\pi}{L} \frac{\Delta\lambda}{\text{FSR}}, \tag{9}$$

where $L$ is the length of the PCM section.

Using this automated procedure, approximately $2\times10^5$ electrical switching cycles and one pair of set/reset optical transmission measurements could be completed within about 22 min.

# 5 C band device

The static switching characteristics of the $Sb_2Se_3$-integrated MZI were characterized using the instrumentation described in Supplementary Section 4. Figure S4(a) shows the transmission spectra of the device in the post-fabrication crystalline state, as well as after subsequent amorphization and crystallization operations. In the initial crystalline state, the MZI exhibits a well-defined interference pattern with an extinction ratio (ER) of 33 dB at a resonance wavelength of 1545.12 nm and a free spectral range (FSR) of 10.3 nm in the C-band.

Upon application of a high-energy pulse (7 V, 20 $\mu$s), the $Sb_2Se_3$ undergoes a transition to the amorphous state, inducing a substantial change in the effective refractive index and resulting in a blue shift of 1.74 nm. The ER increases slightly from 33 to 34 dB, indicating negligible imbalance between the interferometer arms. Meanwhile, the total insertion loss remains nearly constant at 1.1 dB, confirming the low-loss nature and stability of the device during switching.

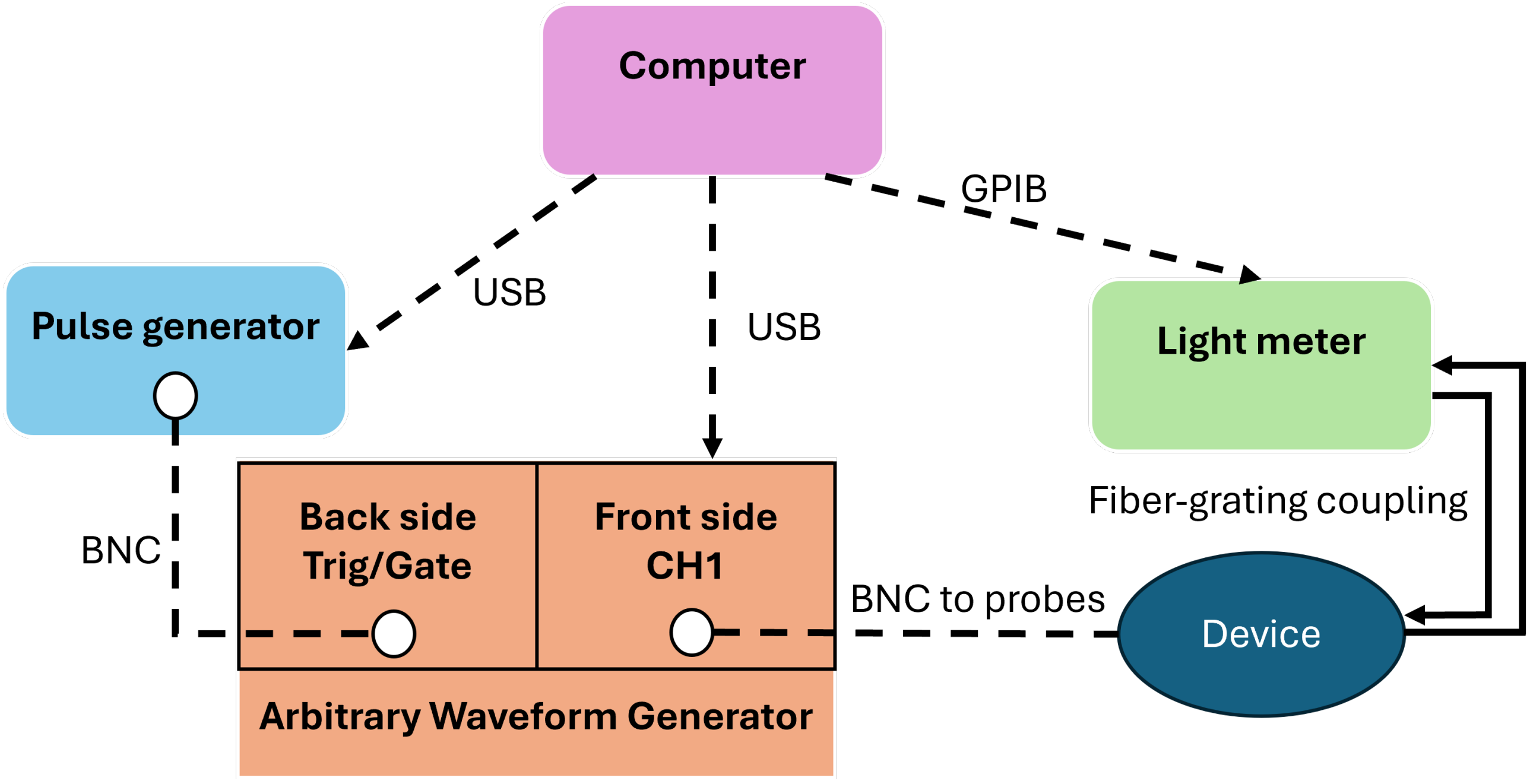


Figure S3: Experiment instruments connection for cycling.

To evaluate reversibility, the PCM was recrystallized using a lower-energy pulse (5.5 V, 600 $\mu$s), resulting in a red shift of approximately 1 nm. This corresponds to a $\sim$57% reduction in phase shift compared to the initial amorphization from the post-fabrication state. For this switching cycle, the maximum ER is approximately 24 dB at 1543.4 nm.

We further investigated the dependence of phase modulation on the PCM length. As shown in Figure S4(b), a clear linear relationship between the induced phase shift and PCM length is observed for C-band MZIs, yielding a maximum phase-shifting efficiency of 0.022 rad/$\mu$m. This value exceeds the maximum phase reported in the Operational Boundaries section of the main text, as the data presented here are obtained from devices with lengths ranging from 10 to 90 $\mu$m, whereas the mapping data are collected from a device of a single length. This discrepancy arises from statistical variations across multiple devices as well as device-to-device variability.

To quantify the contribution of the PCM phase shifter to the overall insertion loss of the MZI, standalone C-band $Sb_2Se_3$ phase shifters were characterized. The propagation losses were extracted as 0.021 dB $\mu m^{-1}$ in the crystalline state and 0.018 dB $\mu m^{-1}$ in the amorphous state. Additional measurements for O-band devices are provided in Supplementary Section 6.

A significant variation in phase-shifting efficiency was initially observed between the post-fabrication crystalline state and the first electrically induced recrystallized state. Identical amorphization (reset) and crystallization (set) pulse profiles were subsequently applied in all following cycles. With continued cycling, as shown in Figure S4(c), the resonance shift gradually decreased and eventually stabilized at approximately 0.004 *to* 0.005 rad/$\mu$m after about $10^6$ cycles. The ER of this 2-bit switching is stable approximately at 6 dB.

This behavior arises because, following fabrication, photonic devices typically undergo an initial stabilization process before reaching reliable operation [13, 14]. The atomic configuration and interfacial conditions of the PCM layer can deviate from an ideal equilibrium state due to residual stress, surface defects, and deposition non-uniformity. Consequently, during the initial switching cycles, device performance, including crystallization kinetics, amorphisation efficiency, and phase modulation depth can exhibit pronounced fluctuations. With repeated set–to-reset cycling, the PCM microstructure progressively evolves toward a quasi-equilibrium state, in which defect populations and atomic vacancies approach a dynamic balance, internal stresses are partially relaxed, and phase boundaries become increasingly well defined and reproducible [15, 16].

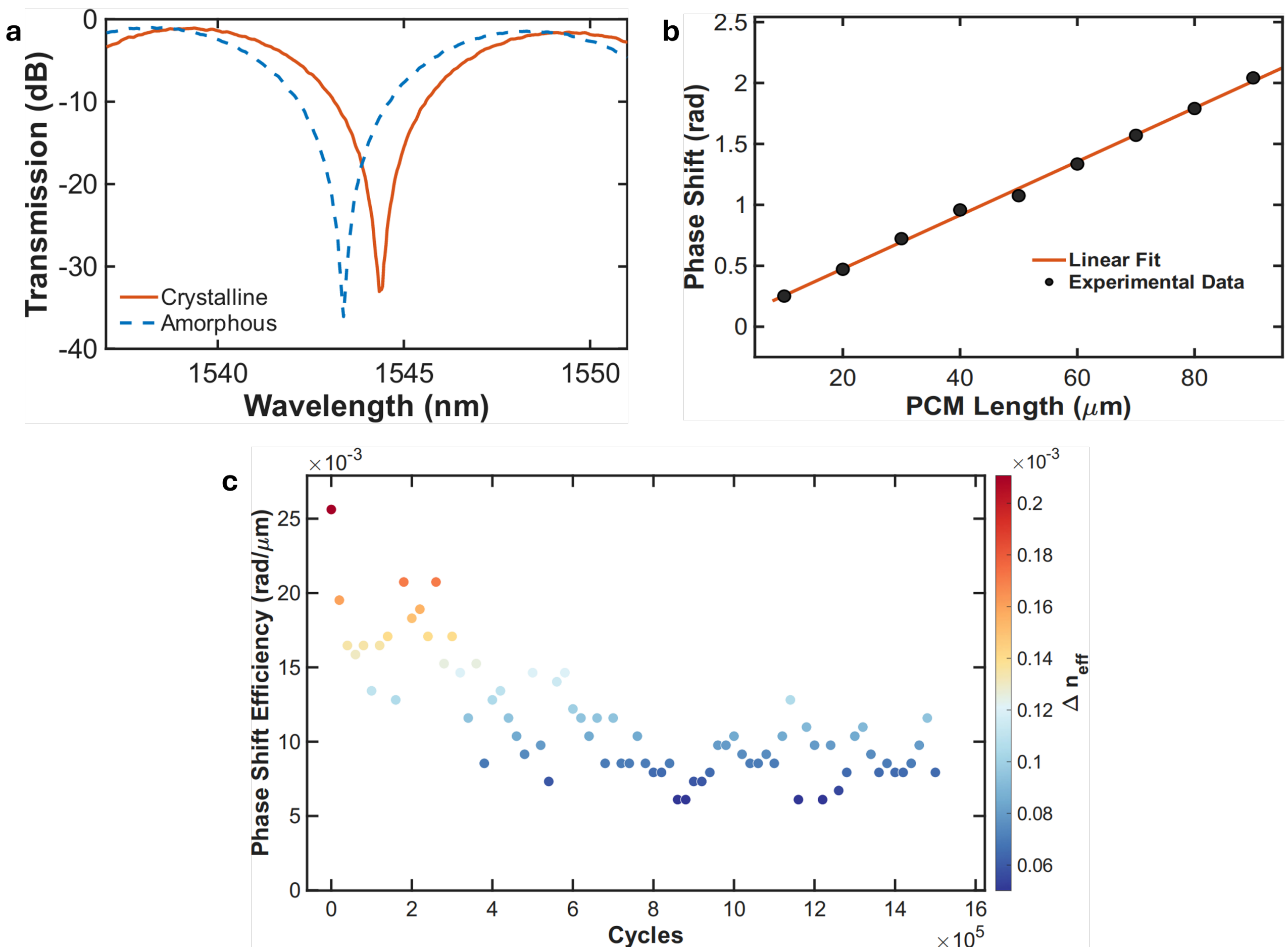


Figure S4: Measurements for C band MZIs. (a) Transmission shift at initial one cycle for a C-band MZI with 30$\mu$m PCM phase shifters. (b) Linear fit of maximum phase change versus PCM length in C-band MZIs. (c) Phase change in initial one million cycles for a MZI with 30$\mu$m PCM phase shifters.

To assess the across-wafer consistency of the fabricated switching devices, the phase-shift efficiency of C-band MZIs was mapped across the wafer, as shown in Figure S5. The measured initial phase-shift efficiency ranges from approximately 0.017 to 0.025 $rad/\mu$m across the measured wafer locations. The phase-shift efficiency is an integrated device-level metric that reflects the combined functionality of the silicon waveguide, electrical heater, PCM, and their thermal and optical interactions. A measurable phase shift therefore requires these constituent components and interfaces to operate properly, enabling the device to perform the intended optical switching function. The observed wafer-level distribution consequently provides an assessment of the reproducibility of the integrated phase-shifting functionality across the wafer.

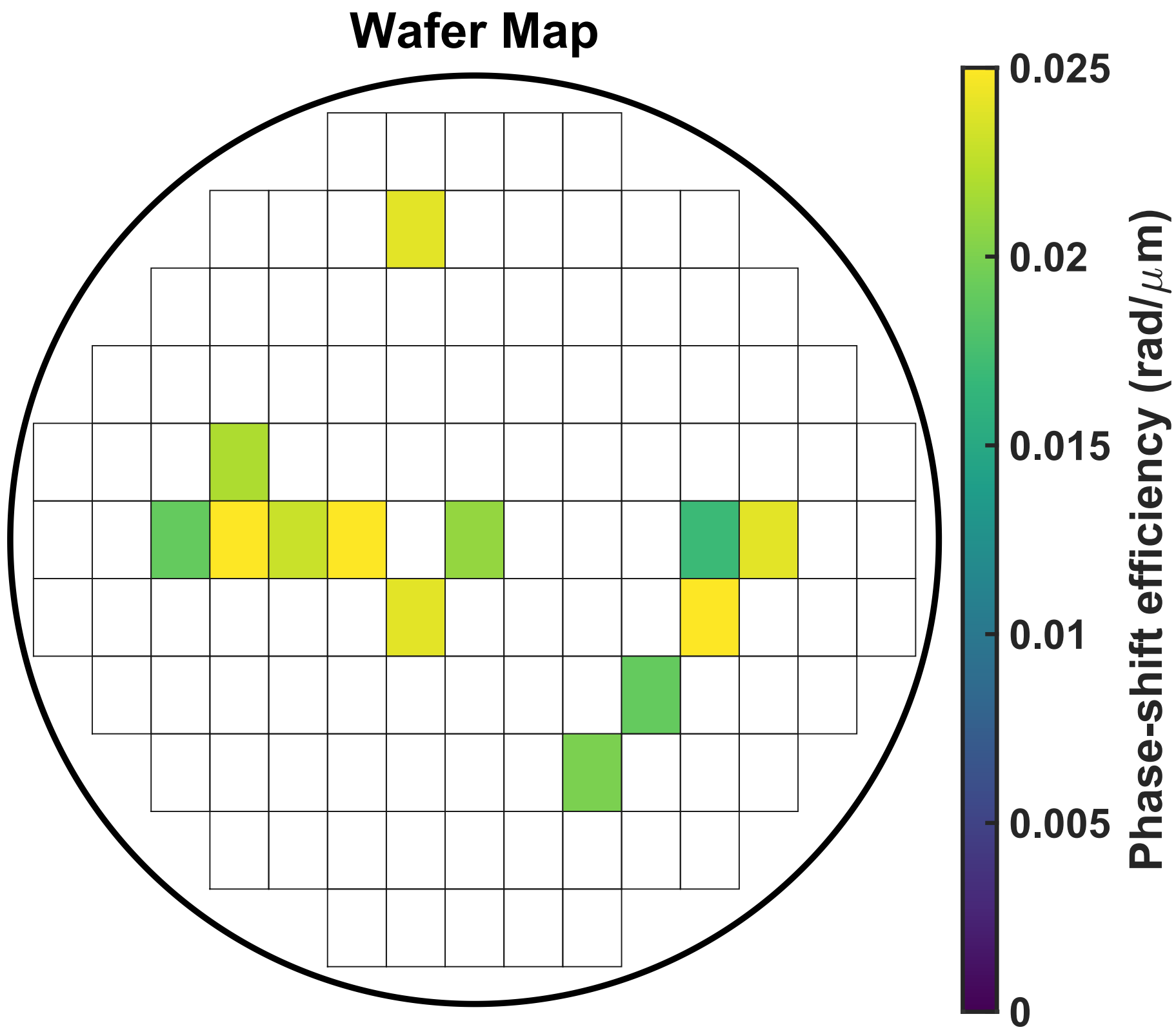


Figure S5: Wafer mapping of the phase-shift efficiency of the C-band MZI devices.

## 6 O band device

Using the same characterization methodology as for the C-band devices, we investigated the initial switching behaviour of O-band MZIs with varying PCM lengths. Figure S6(a) shows the evolution of the transmission spectrum from the post-fabrication state to the first reset state and subsequently to the first set state. A similar behaviour to that observed in the C-band devices is obtained. The total insertion loss of the device is approximately 2 dB, while the ER of this passive structure is around 30 dB for all three states.

Figure S6(b) presents a linear fit of the maximum phase shift as a function of PCM length, yielding a phase-shifting efficiency of 0.017 rad $\mu$m$^{-1}$. The key performance metrics of both O-band and C-band devices are summarized in Table S2.

Table S2: Characterization of MZI Devices at Initial Cycles

| Wavelength | 2-bit ER ($dB$) | Max-ER ($dB$) | Shift efficiency ($rad/\mu$m) | Total IL ($dB/\pi$) |
|---|---|---|---|---|
| O band | 25 | 30 | 0.017 | 3.3 |
| C band | 29 | 33 | 0.020 | 2.5 |

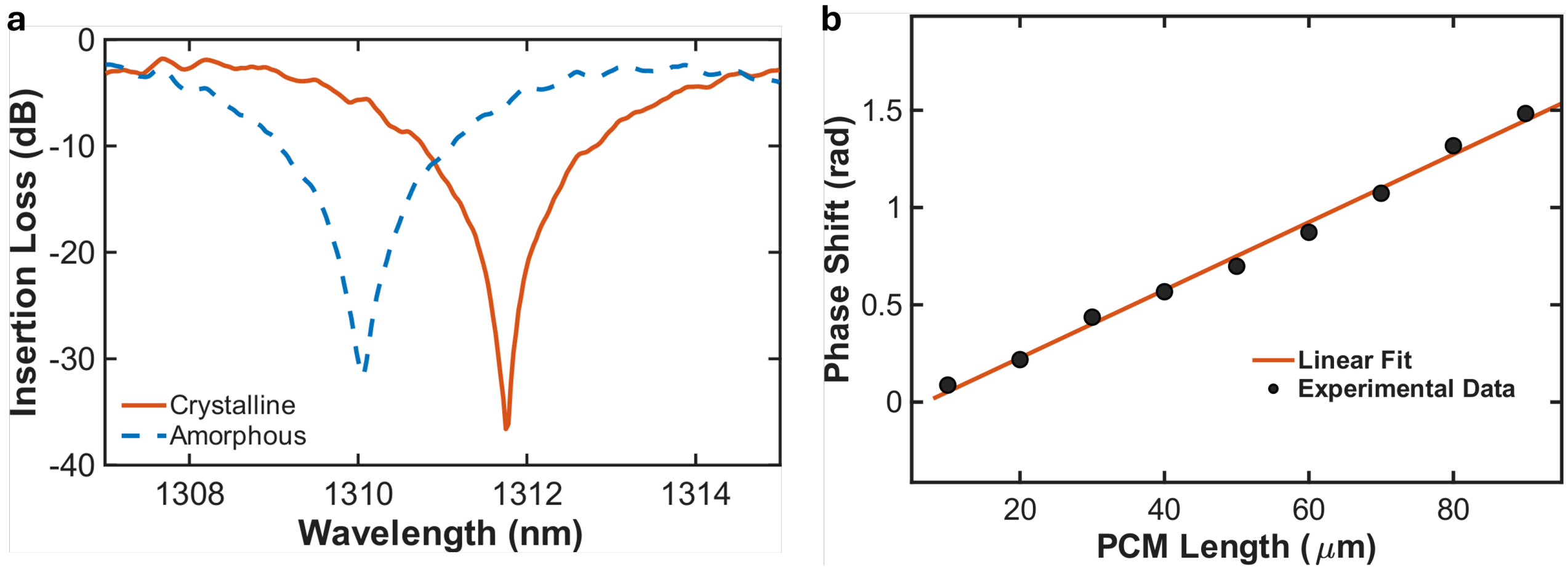


Figure S6: Measurements for O band MZIs. (a) Transmission shift at initial one cycle for an O-band MZI with 90$\mu$m PCM phase shifters. (b) Linear fit of maximum phase change versus PCM length in O-band MZIs.

# 7 Amplify of reset and set pulses during 140 million switching

This section presents the performance of ITO heaters over 140 million switching cycles. The resistivity increases by approximately 40% after 40 million cycles and further rises after 80 million cycles, as shown in Figure S7(a). Figure S7(b) shows the increase in the amplitude of the reset and set pulses required to maintain the peak power for consistent switching conditions.

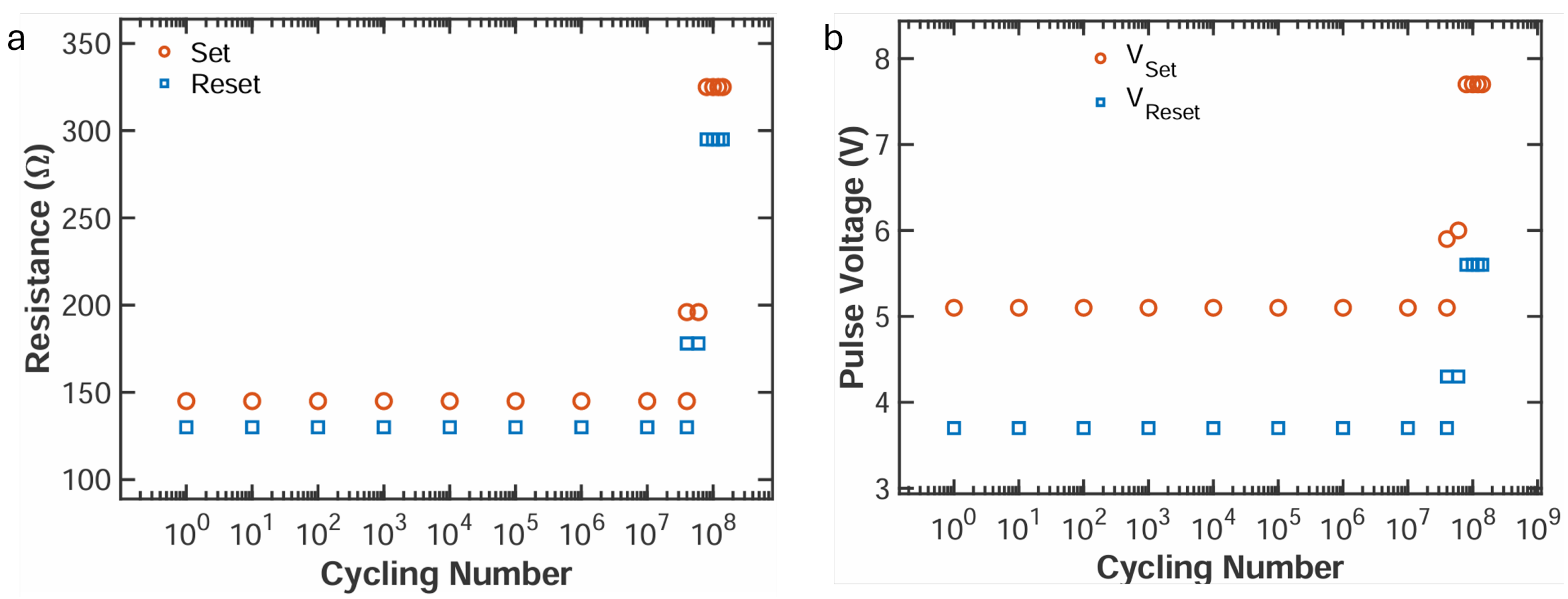


Figure S7: ITO filament performance during 140 million switching cycles. (a) Resistivity of the ITO filament under reset and set pulses over 140 million cycles. (b) Applied voltages of the reset and set pulses on the ITO filament over 140 million cycles.

# 8 Endurance without voltage compensation

In Figure S8, the phase-shift efficiency decreases sharply after approximately 40 million switching cycles. When the device is operated for an additional 40 million cycles under the same electrical driving conditions, the phase shift eventually vanishes. This degradation is attributed to an increase in the resistance of the ITO

heater during prolonged switching. As a result, the peak electrical power delivered to the PCM through the resistive filament decreases, eventually falling below the temperature required to induce the phase transition. Consequently, the applied electrical pulse is no longer sufficient to generate the required temperature rise in the PCM, leading to the loss of the phase-shifting functionality.

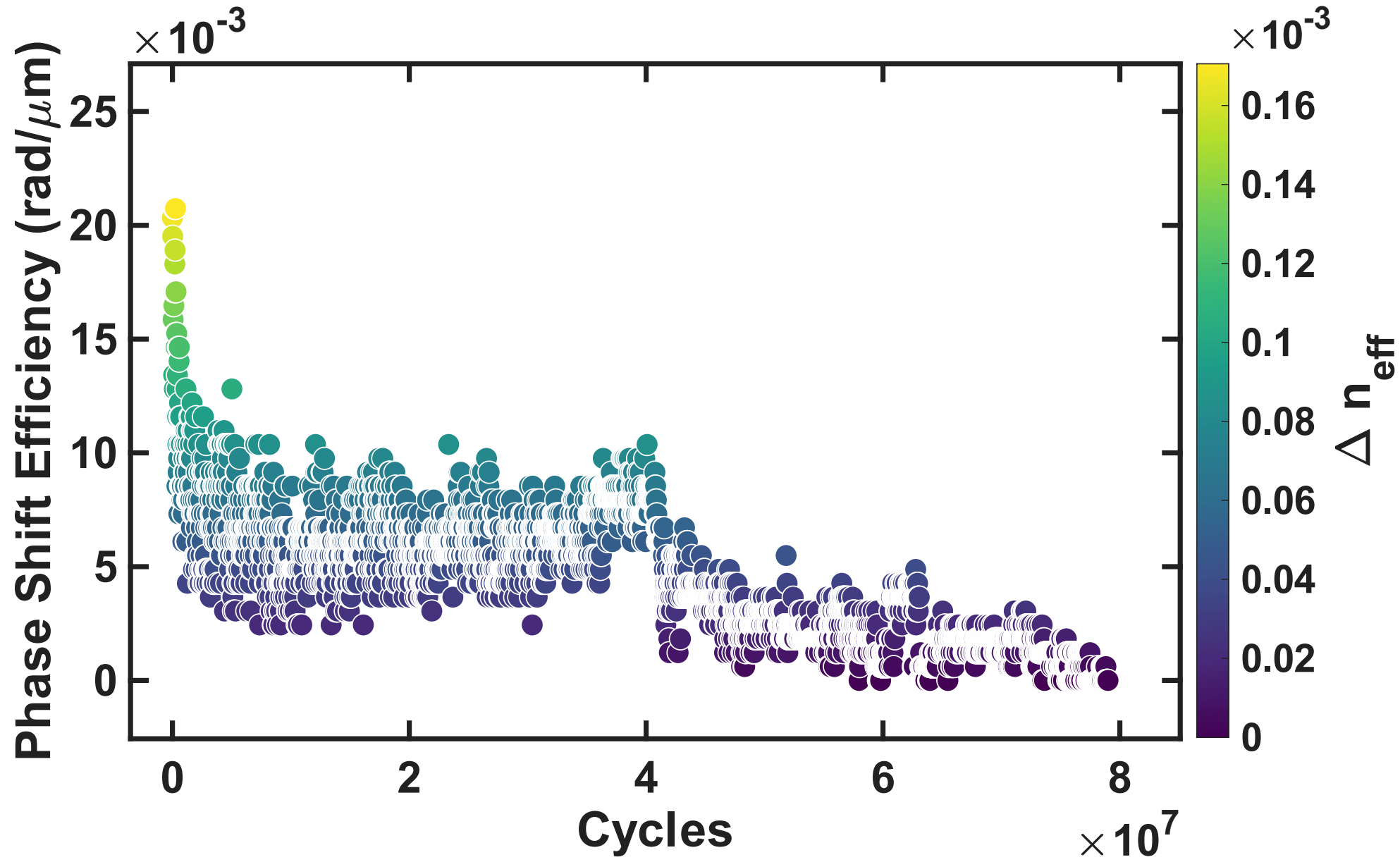


Figure S8: Cycling results under a fixed voltage supply (before 80 million cycles) and the adaptive voltage compensation (after 80 million cycles).

## 9 High-speed transmission measurement

In Figure S9(a), A tunable laser source (TLS) provides the optical carrier, followed by a polarization controller (PC). An arbitrary waveform generator (AWG, Keysight M8199B) generates the electrical waveform, which is subsequently applied to a 65-GHz thin-film lithium niobate (TFLN) intensity modulator (IM). The modulated optical signal is then routed through the photonic switch under test.

At the chip output, a power meter (PM) is used to monitor the transmission loss of the device. The received optical power is adjusted using a variable optical attenuator (VOA). The signal is subsequently amplified by an erbium-doped fiber amplifier (EDFA) and filtered by an optical bandpass filter (OBPF). Finally, the optical signal is detected by a photodetector (PD) and captured by a real-time digital storage oscilloscope (DSO, Keysight UXR1104A) for offline processing.

The digital signal processing (DSP) flow at the transmitter and receiver is illustrated in Figure S9 (b and c). At the transmitter, a pseudorandom binary sequence (PRBS) is generated in MATLAB and mapped onto OOK, PAM4, or PAM8 symbols. The generated waveform is upsampled and pulse-shaped using a root-raised-cosine (RRC) filter with a roll-off factor of 0.2. The signal is then resampled and clipped before being uploaded to the AWG.

At the receiver, the captured waveform is first resampled and then processed using matched filtering, followed by frame synchronization. A decision feedback equalizer (DFE) employing a recursive least-squares (RLS) adaptation algorithm is subsequently applied to mitigate channel impairments. Finally, the bit error rate (BER) is calculated to evaluate the transmission performance.

a

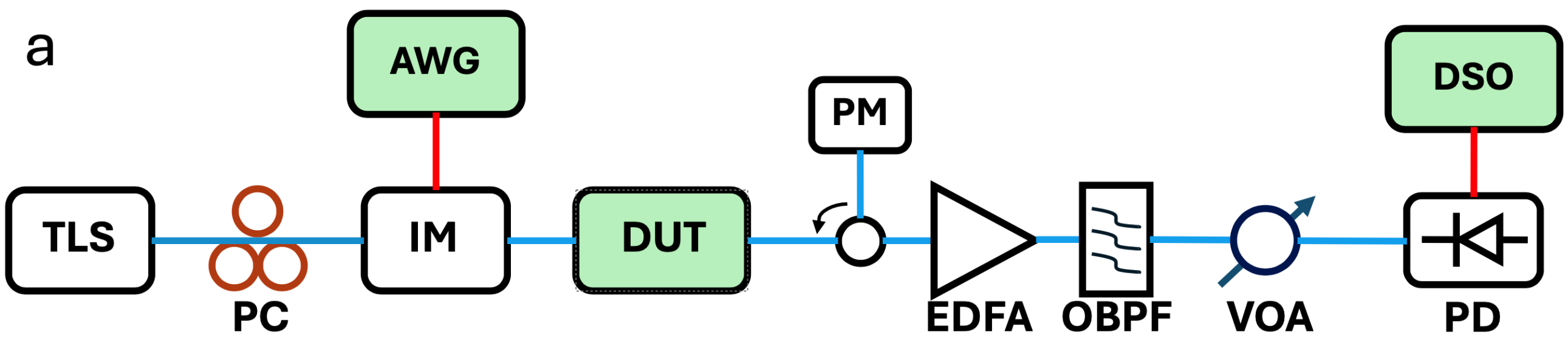


b

| Tx DSP |
| --- |
| Binary Data |
| PAM Mapping |
| Upsampling |
| RRC Filter |
| Resampling |
| Clipping |

c

| Rx DSP |
| --- |
| Resampling |
| Matched Filter |
| Syncronization |
| DFE-RLS |
| BER Measurement |

Figure S9: (a) Experimental setup for high-speed transmission measure-ments. (b, c) Flow charts for the DSP of the M-PAM transmission: Transmitter (Tx) and Receiver (Rx)

# References


(1) Lotkov, E. S.; Baburin, A. S.; Ryzhikov, I. A.; Sorokina, O. S.; Ivanov, A. I.; Zverev, A. V.; Ryzhkov, V. V.; Bykov, I. V.; Baryshev, A. V.; Panfilov, Y. V., et al. ITO film stack engineering for low-loss silicon optical modulators. *Scientific reports* **2022**, *12*, 6321.

(2) Rajendran, V.; Prathuru, A.; Fernandez, C.; Sujatha, D.; Panda, S. K.; Faisal, N. H. Indium tin oxide thin film preparation and property relationship for humidity sensing: A review. *Engineering Reports* **2024**, *6*, e12836.

(3) Ryzhikov, I.; Pukhov, A.; Il'in, A.; Glukhova, N.; Afanasiev, K.; Ryzhikov, A. Anomalous temperature dependence of the conductivity of nanoporous ITO films. *Microelectronic engineering* **2003**, *69*, 270–273.

(4) Li, H.; Guo, L.; Liu, X.; Sun, T.; Li, Q.; Zhang, F.; Xiao, G.; Fu, T.; Chen, Y. High temperature conductive stability of indium tin oxide films. *Frontiers in Materials* **2020**, *7*, 113.

(5) Pruessner, M. W.; Tyndall, N. F.; Walsh, K. J.; Stievater, T. H. Enhanced thermo-optic effects in silicon nitride photonic integrated circuits via polymer claddings. *Journal of Nanophotonics* **2023**, *17*, 046001–046001.

(6) Pierson, H. O. *Handbook of Chemical Vapor Deposition (CVD): Principles, Technology and Applications*, 2nd ed.; William Andrew Publishing: 1999.

(7) Wang, L.; Wen, J.; Yang, C.; Xiong, B. Potential of ITO thin film for electrical probe memory applications. *Science and technology of advanced materials* **2018**, *19*, 791–801.

(8) Yoo, J.-H.; Matthews, M.; Ramsey, P.; Barrios, A. C.; Carter, A.; Lange, A.; Bude, J.; Elhadj, S. Thermally ruggedized ITO transparent electrode films for high power optoelectronics. *Optics express* **2017**, *25*, 25533–25545.

(9) Aryana, K.; Kim, H. J.; Islam, M. R.; Hong, N.; Popescu, C.-C.; Makarem, S.; Gu, T.; Hu, J.; Hopkins, P. E. Optical and thermal properties of Ge2Sb2Te5, Sb2Se3, and Sb2S3 for reconfigurable photonic devices. *Optical Materials Express* **2023**, *13*, 3277–3286.

(10) Pashinkin, A.; Malkova, A.; Mikhailova, M. The heat capacity of solid antimony selenide. *Russian Journal of Physical Chemistry A, Focus on Chemistry* **2008**, *82*, 1035–1036.

(11) Tsu, D. V.; Ohta, T. Mechanism of properties of noble ZnS–SiO2 protection layer for phase change optical disk media. *Japanese journal of applied physics* **2006**, *45*, 6294.

(12) Kim, E.-K.; Kwun, S.-I.; Lee, S.-M.; Seo, H.; Yoon, J.-G. Heat conduction in Z n S: S i O 2 composite films. *Physical Review B* **2000**, *61*, 6036.

(13) Coakley, K. J.; Kabos, P.; Moreau, S.; Obeng, Y. S. Understanding early failure behavior in 3D-interconnects: Empirical modeling of broadband signal losses in TSV-enabled interconnects. *IEEE Transactions on Electron Devices* **2022**, *69*, 6270–6276.

(14) Obeng, Y. S.; Okoro, C. A.; Amoah, P. K.; Dai, J.; Vartanian, V. H. Towards understanding early failures behavior during device burn-in: Broadband RF monitoring of atomistic changes in materials. *ECS Journal of Solid State Science and Technology* **2016**, *5*, N61–N66.

(15) Kim, S.; Burr, G. W.; Kim, W.; Nam, S.-W. Phase-change memory cycling endurance. *MRS Bulletin* **2019**, *44*, 710–714.

(16) Constantin-Popescu, C.; Shalaginov, M.; Yang, F.; Lin, H.-I.; An, S.; Roberts, C.; Miller, P.; Kang, M.; Richardson, K.; Zhang, H., et al. In *Active Photonic Platforms 2022*, 2022; Vol. 12196, pp 26–37.
